# Protons at the speed of sound: Predicting specific biological signaling from physics

**DOI:** 10.1038/srep22874

**Published:** 2016-05-24

**Authors:** Bernhard Fichtl, Shamit Shrivastava, Matthias F. Schneider

**Affiliations:** 1 University of Augsburg, Experimental Physics I, Augsburg, 86159, Germany; 2 Nanosystems Initiative Munich NIM, Schellingstr. 4, 80799 München, Germany; 3 Medizinische und Biologische Physik, Technische Universität Dortmund, Otto-Hahn Str. 4, 44227 Dortmund, Germany; 4 University of Oxford, IBME Old Road Campus Research Building Oxford, OX3 7DQ, UK

## Abstract

Local changes in pH are known to significantly alter the state and activity of proteins and enzymes. pH variations induced by pulses propagating along soft interfaces (e.g. membranes) would therefore constitute an important pillar towards a physical mechanism of biological signaling. Here we investigate the pH-induced physical perturbation of a lipid interface and the physicochemical nature of the subsequent acoustic propagation. Pulses are stimulated by local acidification and propagate – in analogy to sound – at velocities controlled by the interface’s compressibility. With transient local pH changes of 0.6 directly observed at the interface and velocities up to 1.4 m/s this represents hitherto the fastest protonic communication observed. Furthermore simultaneously propagating mechanical and electrical changes in the lipid interface are detected, exposing the thermodynamic nature of these pulses. Finally, these pulses are excitable only beyond a threshold for protonation, determined by the pK_a_ of the lipid head groups. This protonation-transition plus the existence of an enzymatic pH-optimum offer a physical basis for intra- and intercellular signaling via sound waves at interfaces, where not molecular structure and mechano-enyzmatic couplings, but interface thermodynamics and thermodynamic transitions are the origin of the observations.

Significant part of the cells organization origins from membranes. Its basic structure, the so called bilayer, is formed from lipids and incorporates various other biomolecules including enzymes. The common teleological explanation for the existence of such membranes is the regulation of in- and outflux^1^. This article, however, is inspired by the mindset that biological interfaces in general and lipid interfaces in particular are much more than a compartmental element of the living cell. *They are fluctuating thermodynamic systems, which can change state and integrate local actions by propagating waves.* We argue that they are responsive as well as receptive and actively involved in providing specificity for biochemical processes of the cell. The state diagrams of model lipid interfaces similar to a single leaflet of such a bilayer are conveniently recorded using the Langmuir technique^2^,^3^. It allows for a well-defined thermodynamic system where variables, like molecular area *(A)*, lateral pressure *(π)*, temperature *(T)*, surface potential *(ψ)* and pH can be varied quasi-statically over a broad range. The resulting state diagrams present a clear picture of strong thermodynamic couplings observed in these systems, i.e. changes in mechanical properties are inevitably coupled to thermal, electrical and electromagnetic properties of the membrane^4^,^5^,^6^. Interestingly, similar couplings have been observed even during non-equilibrium dynamic processes, in particular, during two dimensional sound propagation in lipid monolayers^7^.

Protons are known to excite gels^8^, plant cells^9^ as well as neurons^10^,^11^ as has been shown in numerous studies. Indeed Konrad Kaufmann has suggested the release of protons as the foundation of synaptic transmission^12^,^13^,^14^. They also play a pivotal role in various signaling pathways. Typically, the function of proteins depends on the surrounding pH with some proteins even exploiting pH gradients, for instance the enzyme ATP synthase^15^,^16^. However, while the effects of equilibrium state of the interface on local protonation kinetics and long range proton conduction via diffusion have been thoroughly investigated^17^,^18^,^19^,^20^,^21^,^22^,^23^, non-equilibrium proton dynamics remains unexplored. In particular with the direct observation of acoustically propagating pulses in lipid interfaces recently published by some of us^24^, pH-pulses seem reasonable and would offer an unprecedented explanation on biological communication and the orchestration of all the individual elements of a cell.

Indeed, here we show that *i)* acoustic pulses can be excited in lipid monolayers through local acidification of the interface, *ii)* that the excitation is specific and exhibits a local pH threshold and *iii)* that the resulting pulse reversibly changes the local pH of the interface. With propagation velocities of ~1 m/s, these pulses are orders of magnitudes faster than the lateral proton translocation at membrane interfaces^18^,^19^ and represent hitherto the fastest “protonic communication” observed. Finally, we discuss the potential of these pulses as a new mechanism for intra- and intercellular biological signaling.

## Results & Discussions

The following section is divided into four parts: At first we will demonstrate that local acidification of lipid monolayers leads to acoustically propagating pressure pulses. Secondly, we will show that the excitation involves head group protonation and thus directly relates to the pK_a_ of the lipid head group, which in turn opens the door for specific excitation. The third part will provide evidence for the adiabatic coupling between pressure pulses and pH of the interface, enabling the local control of pH from remote. In the fourth part these findings will be supported by surface potential measurements, additionally revealing the simultaneous propagation of an electrical pulse. In the final, concluding part we will discuss the biological relevance of these results and propose a new model for specific biological signaling.

### Acidic excitation of acoustic waves

**Figure 1:**
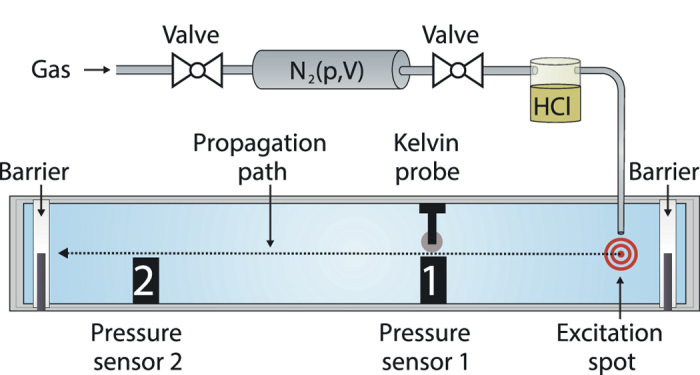
The film balance setup (Langmuir trough) for analyzing propagating monolayer pulses consists of two pressure sensors and a Kelvin probe in order to measure mechanical and electrical changes at the lipid interface. In a typical experiment a fixed amount of nitrogen is blown through a glass bottle partly filled with an acid solution (in this case 32% HCl). The resulting gas mixture is then gently blown onto the lipid monolayer (red spot). Lateral pressure and surface potential are recorded and velocities are calculated. Two moveable Teflon barriers enable us to compress or expand the lipid film and thereby to record lateral pressure and surface potential isotherms. For fluorescent pH-measurements the Kelvin probe is exchanged by an optical setup (not shown). The dyes are excited at 465 nm and the emission is measured at 535 nm and 605 nm (Figure drawn by B. Fichtl).

**Figure 2:**
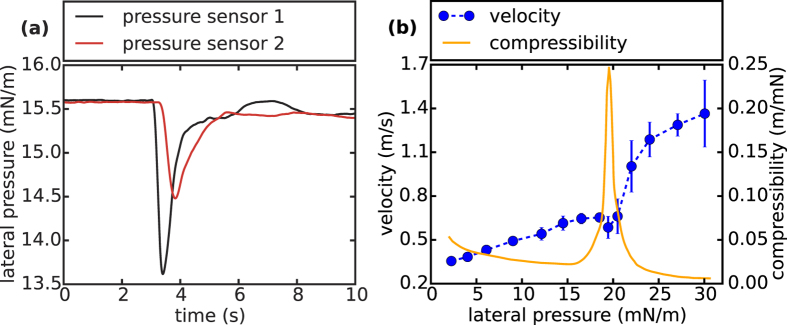
Acidic excitation of acoustic waves: **(a)** Time course of a typical lateral pressure pulse traveling from sensor 1 to sensor 2 in a DMPS monolayer excited by hydrochloric acid. The amplitude of the pulse is only slightly damped (~45%) considering the macroscopic distance of 15 cm. From the time delay between the two pressure changes and the known distance, the propagation velocity can be determined. **(b)** Mean values and standard deviations of pulse velocities of five independent measurements as a function of the lateral pressure *π* of the monolayer at 20 °C. Around 20 mN/m the velocity possesses a distinct minimum. This corresponds to the phase transition pressure of the membrane, which is indicated by the maximal isothermal compressibility *κ*_*T*_ of the interface.

The addition of hydrochloric acid (gas) onto a DMPS monolayer results in a propagating change of lateral pressure [Setup see Fig. 1]. In Fig. 2(a) a typical time plot of the lateral pressure signal *π(t)*, following an excitation by hydrochloric acid gas, is shown. At first the pulse reaches sensor 1, resulting in a strong lateral pressure decrease of around 2.0 mN/m and a relaxation back to equilibrium. Around 0.25 seconds after the first sensor detects the pulse, it reaches sensor 2 with slightly damped amplitude (~45%) considering the macroscopic distance. From the time delay of the pulse between sensor 1 and sensor 2 the propagation velocity *c* can be calculated [Fig. 2(b)]. In the case of a sound pulse *c* should depend on the lateral density *ρ*_0_ and the adiabatic compressibility *κ*_*S*_ of the material. In the linear case^25^:


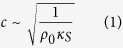


*κ*_*S*_ is not directly accessible, but is may be approximated by the isothermal compressibility^5^,^24^,^26^, obtained from the inverse derivative of the DMPS isotherm:


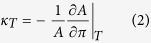


In accordance with its mechanical susceptibility, *c* increases to almost 0.7 m/s in the liquid expanded-phase, followed by a drop to 0.6 m/s in its phase transition regime. At pressures beyond the phase transition region, in the liquid-condensed state, the propagation speed rapidly increases up to 1.4 m/s at 30 mN/m. The correlation between the mechanical properties of the interface and the pulse velocities illustrates the acoustic foundation of these pulses^24^,^27^.

Propagating pulses can also be evoked by other acids, e.g. acetic acid or nitric acid. This indicates the protonic nature of the excitation process, since the only common features between the acids are their dissociated protons [SI–S2]. Furthermore the excitation and propagation of pulses is not limited to DMPS, but also works with other lipids, e.g. DPPG (negatively charged) or DPPC (zwitterionic), demonstrating the universality of the observed phenomenon [SI–S3].

However, not every addition of acid leads to a propagative pulse. There exists a lower and an upper pH threshold for the excitation as described next.

### Subphase pH and specific/threshold excitation

**Figure 3:**
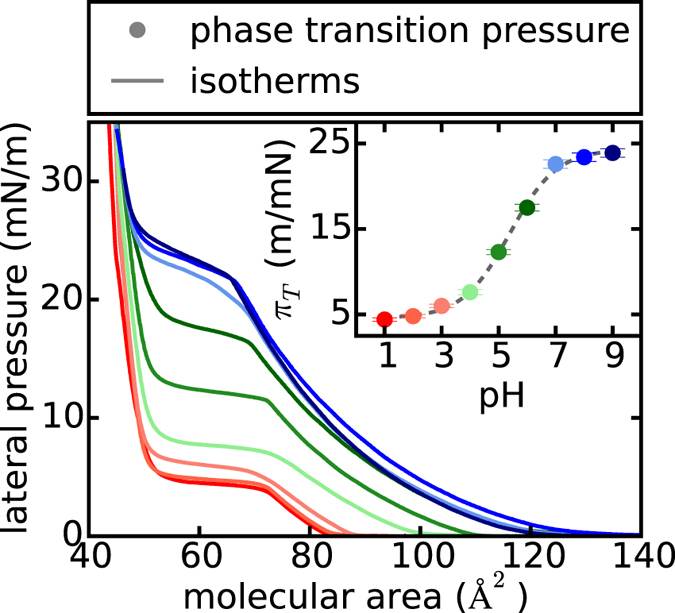
Lateral pressure – area isotherms for varying subphase pH conditions. The plateau regime of the isotherms corresponds to the first order phase transition of the DMPS monolayer. The phase transition pressure *π*_*T*_ increases monotonically in a sigmoidal shape for increasing pH bulk values (See inset). This behavior is typical for the pK_a_-value of the lipid head group. From the first derivate of the sigmoidal fit, we obtain a pK_a_ of around 5.4 which - in good agreement with literature - corresponds to the pK_a_ of the carboxyl group of the lipid (sigmoidal fit: 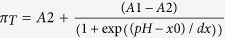 with *A*2 = 24.4mN/m, *A*1 = 4.5mN/m, *x*0 = 5.4 and *dx *= 0.83).

If the subphase of the lipid monolayer is too acidic or too alkaline, no pulses can be excited [SI – S4]. In order to explain the pH bulk dependency of the excitation, the isothermal pH behavior of DMPS is studied [Fig. 3]. The plateau region of the isotherms represents the phase transition of the lipids from the liquid-expanded to the liquid-condensed state. At high pH values (≥7) as well as at low pH values (≤4) the phase transition pressure *π*_*T*_ changes only slightly with pH. In between these two regions, *π*_*T*_ strongly depends on the pH of the subphase, leading to a sigmoidal *π*_*T*_-pH-profile of the lipid. This behavior is well known for charged lipids^28^,^29^ and due to the protonation of the lipid head group. From the first derivate of the curve 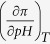, we obtain a pK_a_-value of 5.4 for the carboxyl group of DMPS, in good agreement with literature^28^,^30^.

The dependency of the excitation on the pH of the subphase can now be easily explained by the sigmoidal pK_a_-profile. At high pH values the change in surface pH has to be large enough, in order to facilitate the protonation of the lipids and thereby a detectable propagative change in lateral pressure. If the monolayer is already fully protonated, as it is the case for low pH values, the addition of acid does not lead to propagating pulses anymore. Thus the dynamic response of the interface to a certain excitation 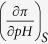, depends to a great degree on its chemical properties and environment, exhibiting a maximum near the pK_a_ of the lipid monolayer. This threshold behavior of the interface introduces “specificity” in the excitation process and allows to control signal strength, which, as described below, opens up new possibilities for “specific communication”.

So far we observed that close to the pK_a_ of the monolayer, local pH changes inevitably lead to lateral pressure changes. Therefore the question arises, if the inverse relationship holds, too: Do propagating pressure pulses evoke pH changes 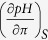 at the interface?

### Propagating pH-pulses

Lipid conjugated fluorescence probes provide a fast, noninvasive and effective method for measuring the local pH at a lipid interface^31^. The emission characteristics of these probes are sensitive to pH changes in its environment, especially near its pK_a_. Importantly, in a lipid monolayer the emission intensity at a certain wavelength is also a function of surface pressure and thus cannot be interpreted in terms of only pH changes^6^. To quantify changes in the optical signal, one is better off measuring the ratio of intensities at two different wavelengths, eliminating the trouble of having to deal with absolute intensities.

**Figure 4:**
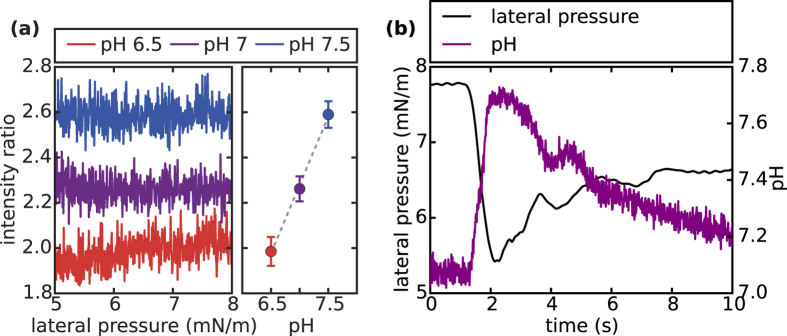
Chemical response of the monolayer to isothermal and adiabatic changes: **(a)** Intensity ratio I_R_ = I_535nm_/I_605nm_ of the fluorescent dye Oregon Green 488 as a function of lateral pressure at different bulk buffer pH-values (6.5, 7 and 7.5). While I_R_ varies tremendously with bulk pH, its dependence on lateral pressure is negligible in the liquid-expanded phase (at least for isothermal expansion). Plotting mean I_R_’s for various bulk pH-values, reveals a linear relationship in the relevant pressure regime (5 mN/m–8 mN/m). This experimental observation enables us to directly translate I_R_ measurements into local pH changes under dynamic conditions, i.e. during the propagation of acoustic pulses. **(b)** Time course of lateral pressure and interfacial pH for a propagating pulse. Clearly, the two detected signals correlate in phase and the pH rises ~ 0.6 units for the given pressure variation. This is the first observation of pH-pulses traveling with the speed of sound along the interface.

Fig. 4(a) shows the intensity ratio I_R_ = I_535nm_/I_605nm_ as a function of lateral pressure between 5 and 8 mN/m during isothermal expansion at different buffer pH values of 6.5, 7 and 7.5, respectively. Clearly, the ratio reacts sensitively to pH changes of the subphase but not to lateral pressure changes at the lipid interface. For a pH increase of one unit from pH 6.5 to pH 7.5 I_R_ increases linearly from 2.0 ± 0.1 to 2.6 ± 0.1. Hence I_R_ can be used as a measure for the local pH and allows for studying possible pH changes at the interface during a propagating pressure pulse. It is important to note, that this behavior is of course a fingerprint of the inherent phenomenology of the specific dye used and *cannot* be generalized. Indeed, without careful calibration in the proper environment a change in intensity cannot be converted into a change in pH. The transfer from bulk to interface for instance can change the dyes characteristics entirely.

Fig. 4(b) depicts the time course of the pH at the dye during a propagating lateral pressure pulse, within the respective “calibration range” of Fig. 4(a). Obviously, the two signals correlate (inversely) and based on the quasi-static coupling [Fig. 4(a)] a pH increase of approximately 0.6 units at the interface takes place. Subsequently the monolayer relaxes back to equilibrium where the pressure as well as the interfacial pH reacquire their former values.

In the same way as proton addition leads to condensation an expansion leads to the liberation of protons from the interface and hence an increase in local pH^29^,^32^. Thus, the negative correlation between pressure and pH origins from the fact, that the propagating front is actually an expansion caused by the local acidification at the excitation site (15 cm distance from the point of detection). These observations suggest that the interface behaves qualitative differently during quasi-static and adiabatic changes: While during quasi-static *isothermal* expansion the pH at the interface remains constant [Fig. 4(a)], it changes substantially during *adiabatic* pulse propagation.

### Electrostatic contributions

**Figure 5:**
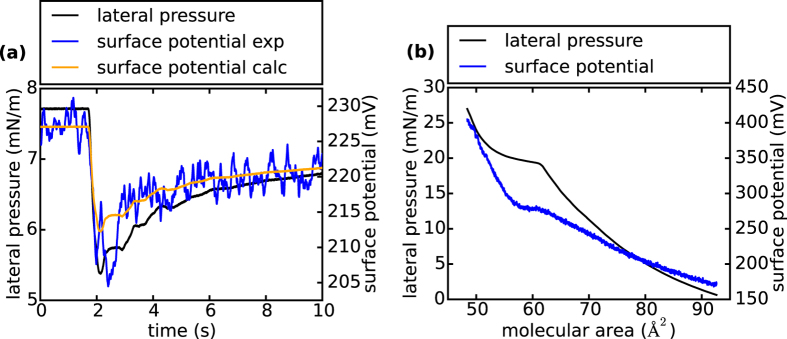
Electrical properties of the DMPS monolayer: **(a)** Readouts of surface potential and lateral pressure during a traveling pulse. The variation of the mechanical signal (~2.4 mN/m) coincides with the variation of the electrical response (~20 mV), elucidating the coupling of all thermodynamic variables even under dynamic conditions. From quasi-static experiments (see Fig. 5b) a surface potential change of ~14 mV can be calculated (surface potential calc), which is ca. 30% less than the measured value (surface potential exp). **(b)** Isothermal measurement of lateral pressure (black) and surface potential (blue) as a function of molecular area. The phase transition of the lipids from liquid-expanded to liquid-condensed phase is clearly visible in both signals (horizontal regimes), conclusively demonstrating thermomechanic-electrical coupling. The concatenation of the two curves leads to a relationship *V*^*total*^ (*π*)(See [SI–S5]), from which the change in surface potential due to a given pressure variation can be calculated.

It is important to note that the propagating pulses are not solely, but *also* mechanical. This is not only obvious from the observed pH-pulse [Fig. 4(b)], but also from measuring lateral pressure *π*(*t*) and total surface potential *V*^*total*^ (*t*) of the interface, revealing a simultaneously propagating voltage pulse [See Fig. 5(a)]. Although the propagating pulse is adiabatic in its nature, a quasi-static approximation from isotherms [Fig. 5(b)] reproduces the pulse in shape and magnitude very well [Fig. 5(a)]. This observation was consistently found to be true for all excitations in the liquid-expanded phase of the monolayer (See [SI–S6]). It seems intuitive that the electrical change at the interface should be closely linked to the measured pH-pulse. In order to calculate this change we follow the work of Möbius and subdivide the total surface potential *V*^*total*^ of the lipid monolayer into two contributions^33^: The hydrophilic interface (head group potential *ψ*) and the hydrophobic interface (tail group potential *V*^*tail*^). Importantly, the pK_a_-value of the head group does not vary for different tail group potentials^33^. This shows that the head group protonation is determined by the head group potential. In order to estimate *ψ*, we need to extract the area change from the measured pressure amplitude of the pulse [Fig. 5(a)] and the isotherm [Fig. 5(b)], using a quasi-static approximation. With this numbers and the known dissociation degree of 88% of the carboxyl groups at pH 7 (*α* = 0.88) [Fig. 3] the head group potential *ψ* follows from the Gouy-Chapman theory (note: the negatively charged phosphate group and the positively charged amino groups compensate each other)^34^,^35^:


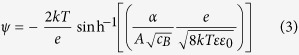


**Figure 6:**
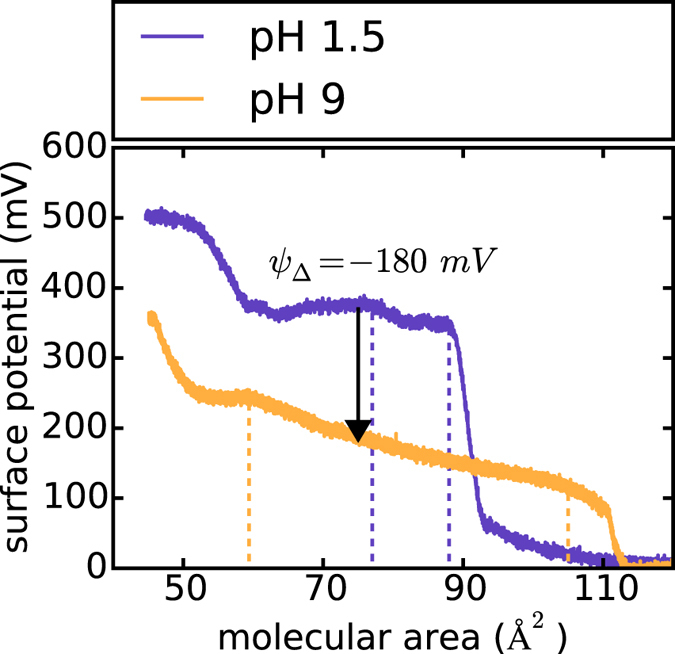
Influence of subphase pH on the surface potential of a DMPS monolayer: A pH change from 1.5 to 9 in the subphase of a DMPS monolayer not only shifts the phase transition pressure to a much higher value [cf. Fig. 3], but at the same time significantly decreases the total surface potential. At a given molecular area the potential of the lipid tails is approximately constant. Therefore the change in the total surface potential from pH 1.5 to pH 9 can be related to the change in head group potential *ψ*_Δ_ during the protonation process of the carboxyl groups. For an area of 75 Å^2^: *ψ*_Δ_ = −180 mV. The start and the end of the liquid-expanded phase are marked by the dashed lines, respectively.

For an area change of A_1_ = 75 Å^2^ to A_2_ = 82 Å^2^, we obtain 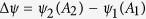 = −113 mV + 117 mV = +4 mV. That is, the propagating expansion front locally increases the head group potential, which will lead to a proton release from the surface^32^. The reliability of this calculation can be tested experimentally from surface potential measurements at different subphase pH-values [See Fig. 6]. At pH 1.5 the carboxyl groups are completely protonated, while at pH 9 they are entirely ionized. At one certain molecular area the contribution of the tail groups to the total surface potential should be constant. Hence when building the difference between two states of protonation at a given area, the tail group potential drops out:


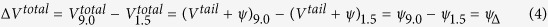


The observed deprotonation leads to a change of 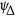 = −180 mV at an area of 75 Å^2^ [See Fig. 6]. Taking into account the dissociation degree of 0.88 at pH 7 [Fig. 3] 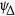 = −158 mV, which is in good agreement with the surface potential of the Gouy-Chapman theory. In order to get a quantitative estimate of the interfacial pH change the Boltzmann distribution can be used (*I* ≙ interface, *B* ≙ bulk)^29^:


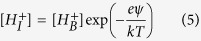


For a surface potential change from *ψ*_1_(*A*_1_) to *ψ*_2_(*A*_2_) equation 5 gives: ΔpH_I_ = +0.1. This qualitatively yields the right trend (the surface pH increases), although it somehow underestimates the measured change of 0.6. The discrepancy between the two values is not totally clear, however, differences ought to be expected, for instance, from the differences in adiabatic and isothermal behavior of the system. What is more, our approximation neglects the decrease of the pK_a_-value of the carboxyl group due to the expansion of the interface^29^,^32^.The shift in pK_a_ will further decrease the proton affinity of the lipid molecules and thus enhance the observed effect^36^. Finally, although we carefully calibrated the fluorescent probe, the propagating electric field may also interfere with the emission.

We would like to add, that for stronger excitations we could record pulses with amplitudes >100 mV [SI – S7]. This is well in the order of action potentials and should be considered during the current controversial discussion on the underlying mechanism of action potentials^26^,^37^,^38^.

In summary, we imagine the electrical pulse to consist of two contributions: One from the air/tail interface and one from the head group/water interface, of which only the latter affects the local pH. The change in the hydrophobic part is therefore: *V*^*tail*^ = *V*^*total*^ − *ψ*. The electrostatic measurements as well as the surface pH measurements lead us to a consistent interpretation: a pH increase at the interface during the adiabatic pulse. We do not attempt to provide an explanation for the observed discrepancy between isothermal and adiabatic response, as this is rather the rule than the exception and already known from the ideal gas. Since mechanical, electrical and chemical properties within this system are coupled, it comes with no surprise that the electrical and chemical response vary as well from isothermal to adiabatic expansion. Nevertheless, we imagine that important insights on the internal timescales of the system may arise from measurements where local processes can be monitored with high time resolution. FCS seems to be the proper tool to open up this door.

## Conclusion

We have shown that lipid monolayers enable propagating mechano-chemical-electrical pulses with velocities controlled by the compressibility of the monolayer. The acoustic waves can be evoked only above a certain threshold. This threshold origins from a transition, namely the head group protonation and is hence determined by the pK_a_ of the lipid interface.

**Figure 7:**
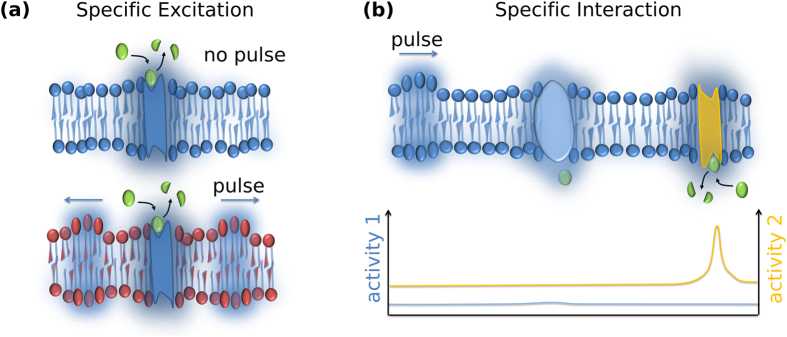
Specific acoustic communication at biological interfaces: **(a)** Specific pulse excitation: An enzyme creates a local change in proton concentration. Only if the resulting local pH ~ pK_a_ of the lipids a pulse is excited. This scenario is illustrated in the lower part of the figure, where the lipids (red) are susceptible to the enzyme-induced pH change. As a result pulses propagate along the interfaces (indicated by blue arrows). In the upper part, the pK_a_ of the surrounding lipids is too acidic and the enzyme-induced change in local pH is insufficient to evoke a protonation transition (i.e. crossing the local pK) and hence a propagating perturbation. **(b)** Specific protein interaction: Enzymes exhibit a maximum activity at a certain pH (pH_opt_). Only if the propagating pH-pulse carries the enzyme environment into or out of the pH_opt_ regime, significant pulse-enzyme interaction is observed and the enzyme can be either “switched-on” or “off”. In this cartoon the activity of the yellow enzyme is switched on, while the activity of the blue enzyme is hardly affected by the pH-pulse (blue and yellow enzymes have different pH_opt_). The interplay between specific excitation depending on the pK_a_ of the interface and the specific interaction depending on the pH_opt_ of the enzyme results in specific signaling between two enzymes. Of course, coupling of pulses to proteins can also take place electrically via charged groups or mechanically and are expected to be particularly increased near the lipid phase transition (Idea from MFS, Figure drawn by SS and BF).

It is important to point out, that the propagation of local pH perturbations as described follows from fundamental physical principles applied to the phenomenology of interfaces. Providing significant localized proton release, it has therefore to be expected to exist in biology as well, even if velocities and/or amplitudes may vary significantly. Previously we have therefore proposed acoustic pulses as a new physical foundation for biological communication^24^,^26^. The results presented here constitute a crucial step in confirming this speculation as they *i)* begin to bridge the gap between physics (adiabatic pulses due to protonic transitions) and biochemistry (regulation of enzymes) and *ii)* introduce a thermodynamic concept of *specificity* [Fig. 7]. In the following we are taking the liberty to briefly outline our ideas. We imagine an (membrane-bound) enzyme, which – as we will explain - will first serve as stimulus and in the next step as receptor for specific pulses:

### Specific excitation

In its catalytically active state many enzymes (e.g. esterases, lipases) will locally liberate protons^39^,^40^. If the proton concentration and hence the pH reaches a certain threshold and if the protonation of the lipids proceeds fast enough, a propagating sound pulse will be triggered [Fig. 7(a)]. Its amplitude depends on the pK_a_ of the interface and on the strength of the excitation. Specificity comes in through transitions: the protonation transition (pK_a_) and potentially order-disorder transition of the lipid tails.

### Specific Interaction

Due to its mechanical, electrical and in particular chemical properties, the propagating pulses will affect proteins at the interface (e.g. enzymatic activity) in the same way it changes the emission properties of the dye here. In Fig. 7(b) two possible interactions of a pH-pulse with an enzyme are shown: If the local pH (pH_loc_) is far from the enzyme’s pH optimum (pH_opt_), the pH-pulse will have only minor impact on the enzyme’s activity. If, however, the surrounding pH is close to the pH_opt_ of the enzyme, the enzyme activity could change enormously: Increasing, if the local pH is shifted towards pH_opt_ or decreasing when the local-pH is shifted away, i.e. (pH_loc_ − pH_opt_) decreasing or increasing, respectively.

Taken together, only if *i)* the stimulus of enzyme A leads to a propagating pulse across the interface and *ii)* the pulse shifts the local pH at enzyme B towards or away from its pH_opt_, effective and specific communication between enzyme A and B will take place.

*It has to be stressed, that in contrast to earlier models, here specificity arises from two (nonlinear) transitions and thus from physical principles rather than structural considerations*. This also implies, that the mechanism of enzymatic regulation is not simply mechano-enyzmatic coupling, but is thermodynamic in nature and “exploits” the existence of transitions (order-disorder as well as protonation transitions). Clearly, specificity can be further enhanced if nonlinear relations between activity and other physical parameters, e.g. compressibility, heat capacity, electrical capacity etc. exist. Such relations have indeed been observed extensively and the maximal activity of phospholipase A_2_ and phospholipase C at the lipid phase transitions are excellent examples^40^. Along the same lines it may turn out that only nonlinear (e.g. solitary) waves are sufficiently strong in amplitude to induce changes at a distance remote from the excitation. We have shown, that interfacial solitary waves create 10–100 fold stronger local changes in pressure and voltage when compared to linear waves^26^. The existence of such solitary waves, however, requires a range of specific conditions, for instance the vicinity of a phase transition as well as a threshold strength of excitation. It seems obvious, that the simultaneous appearance of solitary waves, local protonation-transitions represent a very unique and rare combination of conditions, which would make the proposed enzyme-enzyme communication a very specific process. *Importantly, this follows from thermodynamic and does not require structural information.*

It will be thrilling to see whether the type of communication suggested can be verified as a fundamental principle to orchestrate the individual elements of a cell, cell clusters or even entire organs. Experiments along the lines of those in^44^,^45^,^46^,^47^ will have to show.

## Methods

1,2-dimyristoyl-sn-glycero-3-phospho-L-serine (DMPS), 1,2-dihexadecanoyl-sn-glycero-3-phospho-(1′-rac-glycerol) (DPPG) and 1,2-dipalmitoyl-sn-glycero-3-phosphocholine (DPPC) were purchased from Avanti Polar Lipid (USA) and used without further purification. Monolayers were spread from a chloroform/methanol/water solution on a customary Langmuir trough (NIMA) until the desired lateral pressure was achieved. Measurements were started 10 minutes after solvent evaporation. If not further specified, all measurements were performed at 25 °C on a buffer solution (pH 7.0) containing deionized water (resistivity >18 MΩcm), 100 mM sodium chloride, 10 mM phosphate buffer.

The Langmuir trough is equipped with two Wilhelmy plate pressure sensors, situated 15 cm apart from each other and a Kelvin probe sensor, facing pressure sensor 1 [Fig. 1]. The rapid readout of the sensors (10000 samples/s, 0.01 mN/m and 5 mV resolution) ensures accurate velocity and surface potential measurements. For the detection of fluorescent signals the Kelvin probe is substituted by an optical setup (not shown).

Pulses are excited blowing a fixed amount of pure nitrogen gas (5 ml for sole lateral pressure measurements and 25 ml for surface potential and pH measurements) through the gas phase of a glass bottle filled with 32% hydrochloric acid solution (for reference measurements: 100% acetic acid). pH measurements show that the excitation by 25 ml nitrogen gas drags along (2.0 ± 0.2) × 10^−6 ^mol of hydrochloric acid. The acid/nitrogen gas mixture is then gently blown onto the lipid monolayer in order to prevent capillary waves. The excitation takes place 10 cm away from pressure sensor 1. The gaseous excitation allows for protonating bigger areas of the monolayer while using less amounts of acid than it would be possible for pipetting.

To exclude artifacts, we performed reference measurements on pure water surfaces. Neither nitrogen, nor hydrochloric acid or acetic acid induced any detectable pressure change at the surface. Furthermore pure nitrogen gas was blown onto a DMPS monolayer, to exclude any excitatory effect by N_2_ [SI–S1].

pH changes at the interface are detected using lipid conjugated pH sensitive dye Oregon Green^®^ 488 1,2-Dihexadecanoyl-sn-Glycero-3-Phosphoethanolamine spread along with DMPS (1 mol % dye). The emission of the dyes embedded in the monolayer was measured at 535 nm and 605 nm simultaneously with lateral pressure. Propagating changes were measured at a distance of 10 cm from the excitation spot. In order to rule out diffusion effects, a Teflon ring with a small opening facing away from the excitation spot was used to encircle the lipid monolayer around the spot for optical measurements.

## Additional Information

**How to cite this article**: Fichtl, B. *et al.* Protons at the speed of sound: Predicting Specific biological signaling from physics. *Sci. Rep.* **6**, 22874; doi: 10.1038/srep22874 (2016).

## Supplementary Material

Supplementary Information
