# Supplementary material for: Protons at the speed of sound: Predicting specific biological signaling from physics
Source: Sci Rep. 2016 May 24;6:22874. doi: 10.1038/srep22874 (PMC4877590; doi:10.1038/srep22874)
Supplement: Supplementary Information [file srep22874-s1.doc]

**Supporting Information**

**Title:** Protons at the speed of sound: Predicting specific biological signaling from physics

**Author list:**

Bernhard Fichtl, Shamit Shrivastava, Matthias F. Schneider


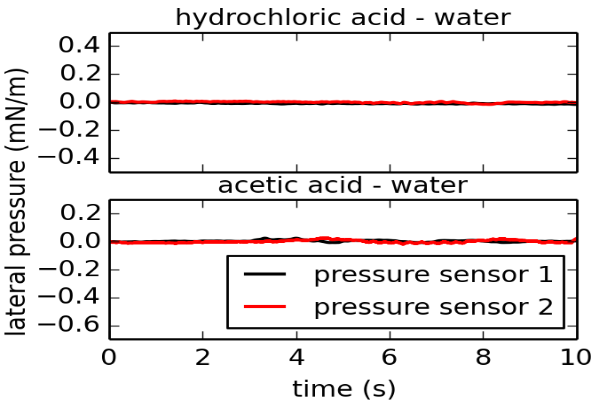

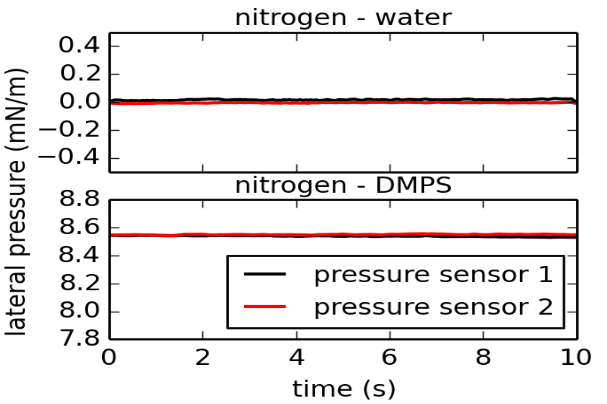


**S1:** Reference measurement on a pure water surface. None of the excitations (hydrochloric acid, acetic acid and nitrogen) are able to evoke any measurable pressure change and hence propagating sound waves on the pure water surface (excitation at ~3 s). Moreover nitrogen does not possess any excitatory effect on DMPS monolayers, too. Consequently all excited pulses in our monolayer experiments have to be due to the interaction between the lipids and the acids (protons) (25°C, 100 mM NaCl, 10 mM phosphate buffer, pH 7.0).


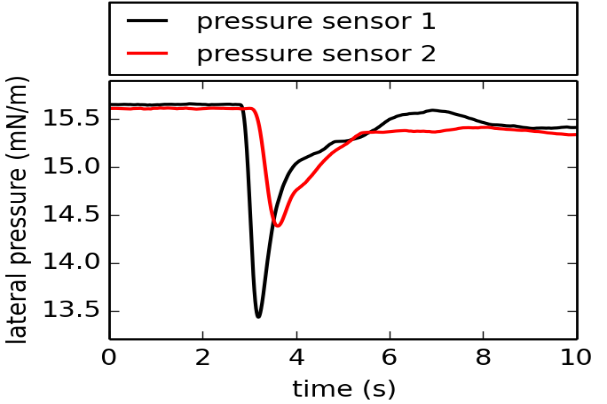


**S2:** Excitation of a propagative pulse across a DMPS monolayer by acetic acid. The time course of the pulse is strikingly similar to the hydrochloric excitation [see Figure 2(a)]. The only common denominator between these two different acids are their protons, elucidating the protonic nature of the excitation (25°C, 100 mM NaCl, 10 mM phosphate buffer, pH 7.0).


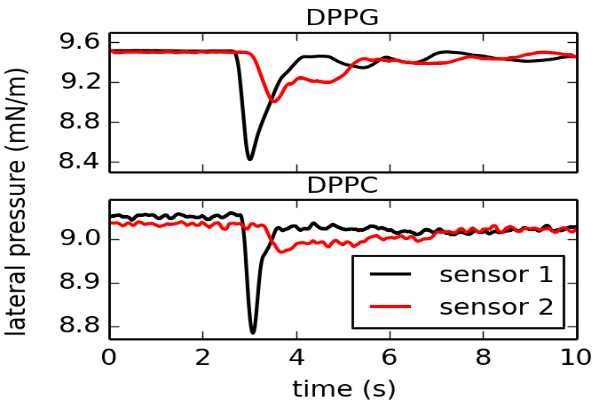


**S3:** Time course of the excitations of DPPC (bulk pH = 3.0) and DPPG (bulk pH = 7.0) monolayers by hydrochloric acid. Surprisingly, pulses can be induced in uncharged (zwitterionic) lipid monolayers like DPPC, too, although with a much smaller amplitude than in charged lipids like DPPG. These experiments illustrate the universality of the pH-driven pulses in lipid systems (25°C, 100 mM NaCl, 10 mM phosphate buffer).


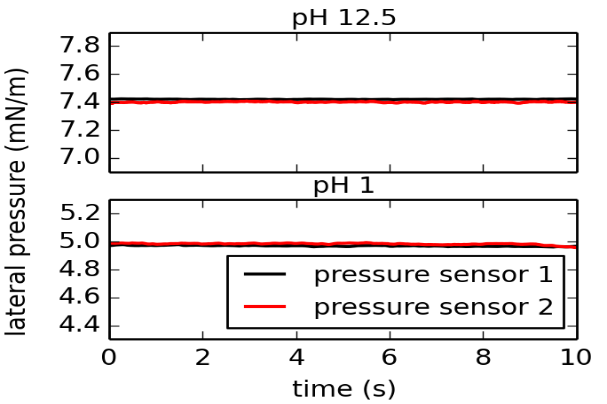


**S4:** At very high, respectively low pH-values (pH 12.5, pH 1), the DMPS monolayer cannot be excited by HCl (excitation at ~3 s). The reason is the pK_a_-profile of the lipid head group [see Figure 3]. At low pH-values the lipids are already protonated, while at high pH-values the excitation has to be strong enough in order to facilitate the protonation of the fully deprotonated head groups (25°C, 100 mM NaCl, 10 mM phosphate buffer).


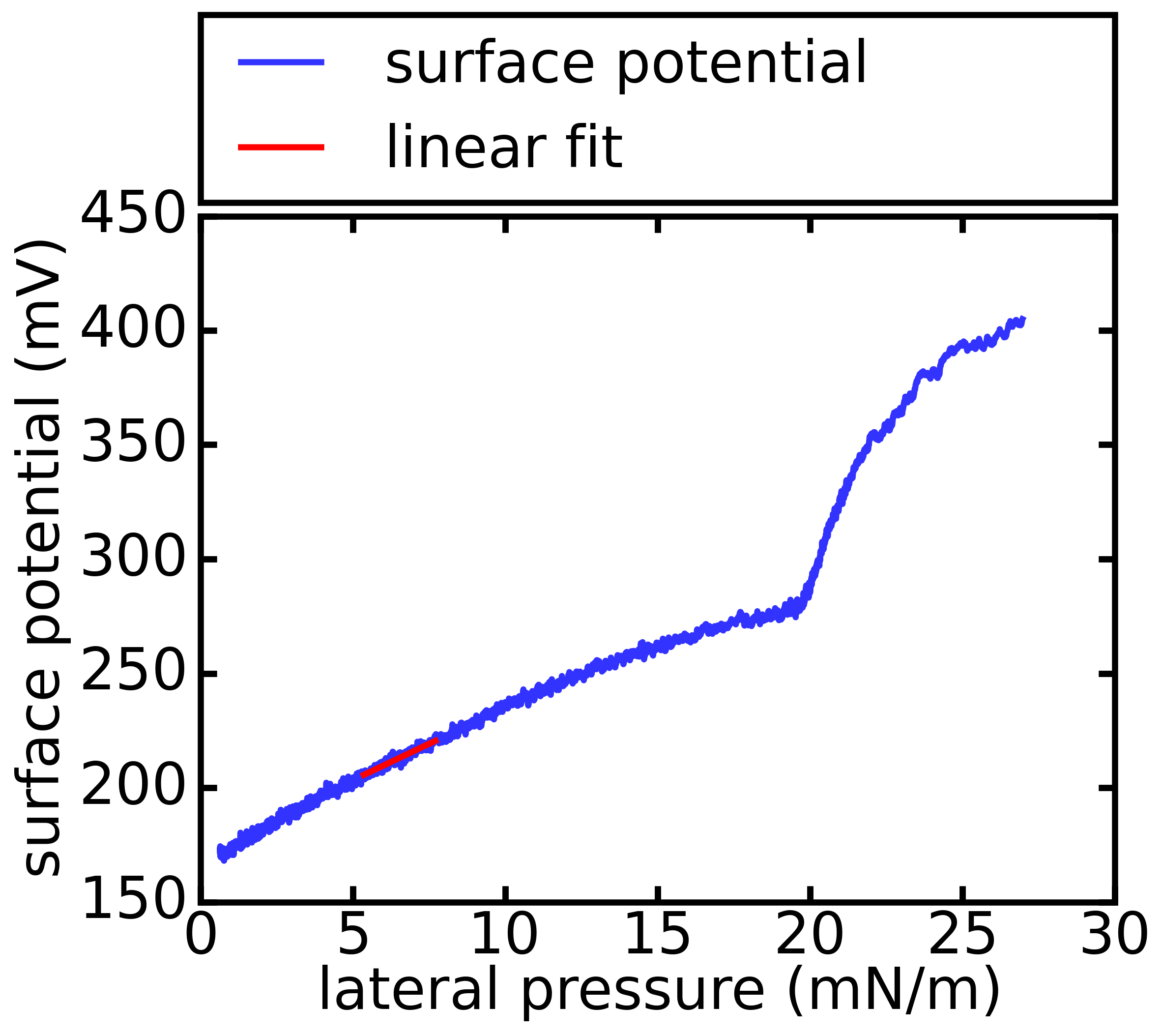


**S5:** Concatenation of lateral pressure and surface potential $\Delta V(\pi)$ extracted from Fig. 5a. The linear fit enables the calculation of the (isothermally) expected electrical response of the monolayer due to the given pressure variation from ~7.7 mN/m to 5.3 mN/m in Fig. 4b (20°C, 100 mM NaCl, 10 mM phosphate buffer, pH 7) (linear fit function: $\Delta V=m\cdot\pi+t$ with $m=6.3$mV/(mN/m) and $t=298.1$ mV).


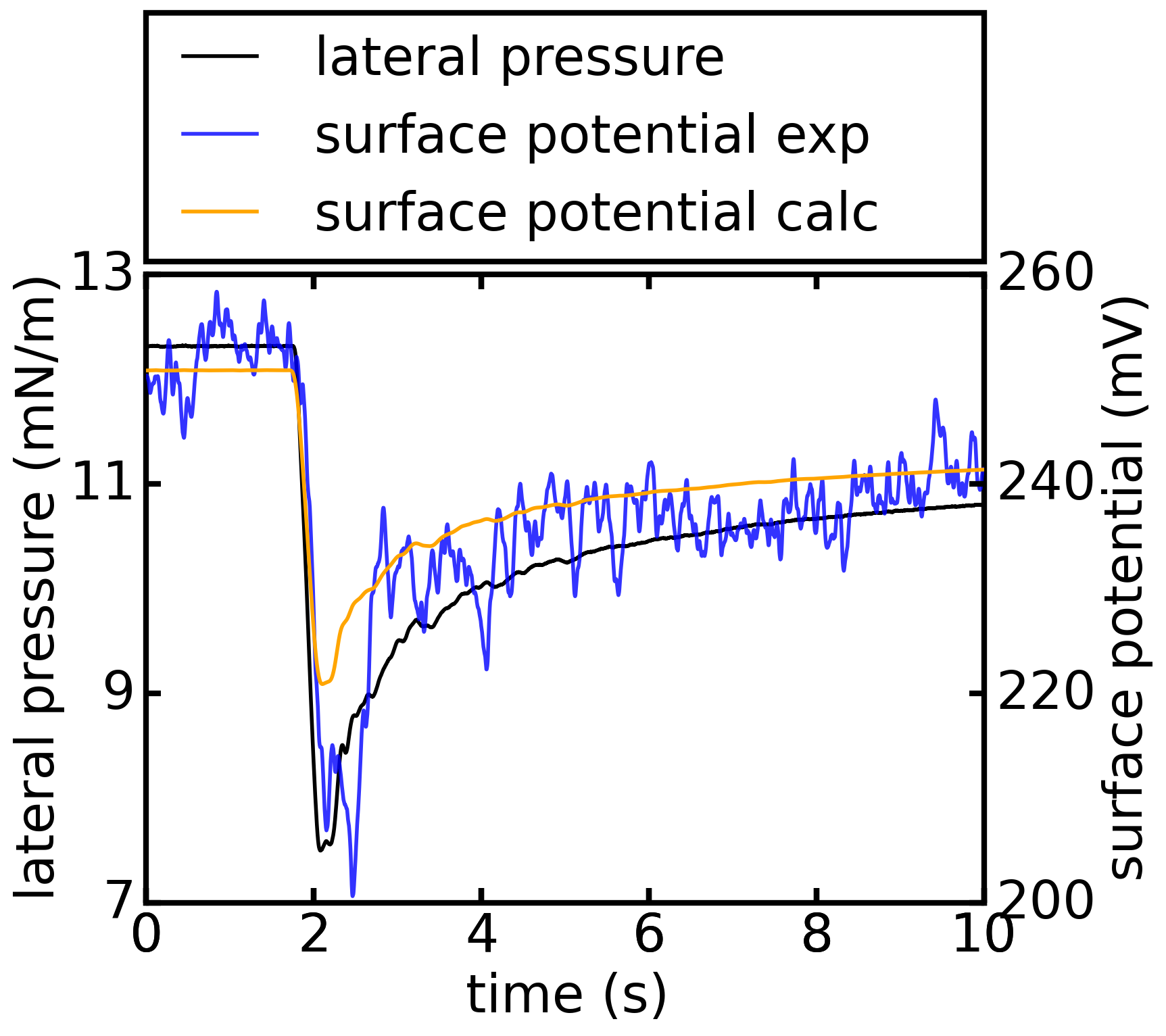


**S6:** Typical time course of surface potential and lateral pressure for a propagating pulse. Mechanical and electrical response of the monolayer are coupled in phase and amount to ~4.8 mN/m and ~50 mV, respectively. The calculated surface potential change (surface potential calc), which is expected for the given pressure change from isothermal couplings (see S5-SI), is considerably smaller than the experimental value (surface potential exp).


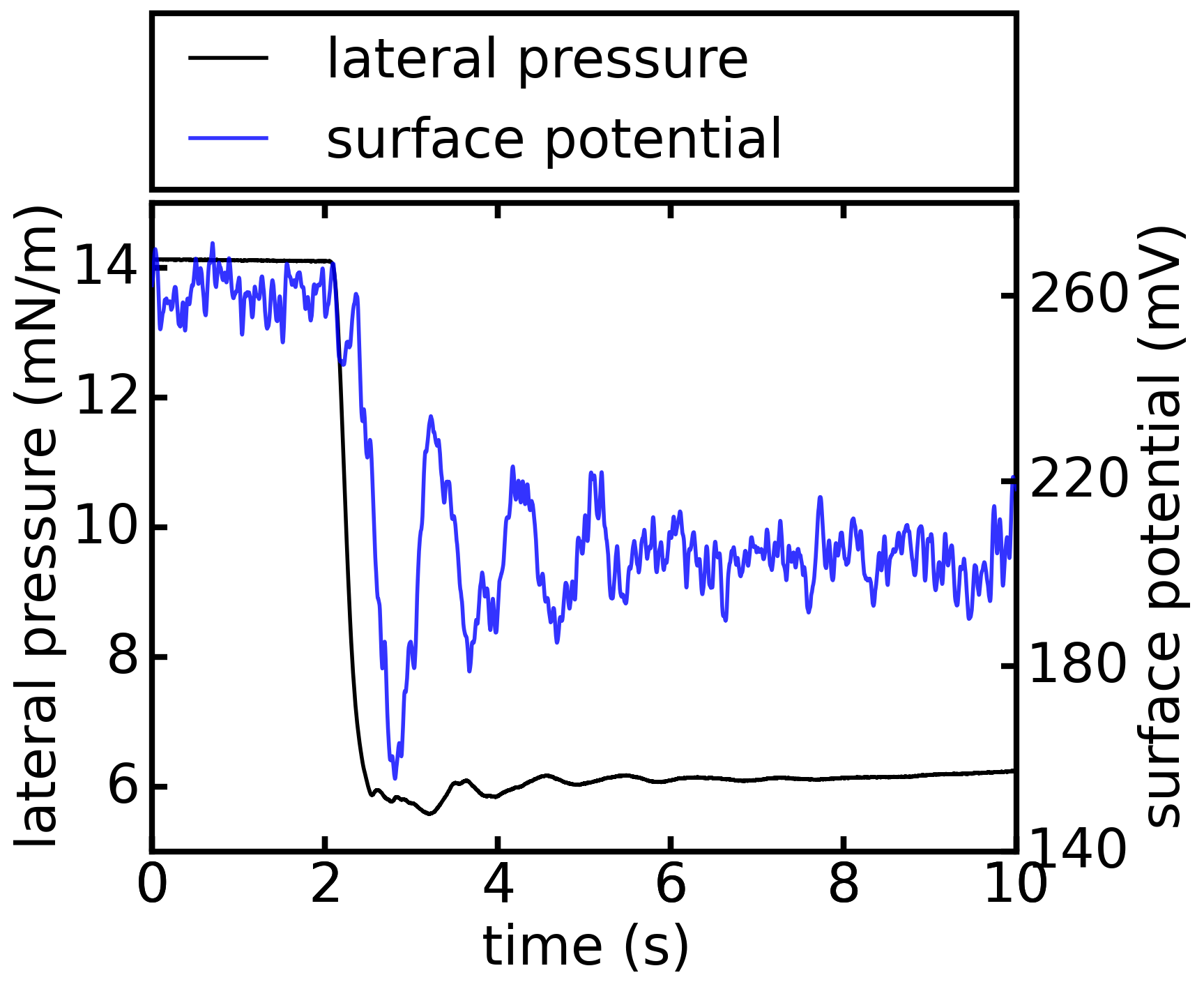


**S7:** For strong excitations and/or less buffer concentrations, pulses with amplitudes >100 mV can be observed. “Strong excitations” are induced by blowing 50 ml nitrogen gas through the gas phase of a 32% hydrochloric acid solution (25°C, 10 mM NaCl, 1 mM phosphate buffer, pH 7). This leads to electric field variation during a pulse of the order of 10^5^ V/m across the surface. The evoked pulses are travelling across the whole interface and get reflected at the end of the film balance. This leads to the visible oscillations in lateral pressure and surface potential.
